# Distinct Patterns of Wnt3a and Wnt5a Signaling Pathway in the Lung from Rats with Endotoxic Shock

**DOI:** 10.1371/journal.pone.0134492

**Published:** 2015-07-28

**Authors:** Hiong-Ping Hii, Mei-Hui Liao, Shiu-Jen Chen, Chin-Chen Wu, Chih-Chin Shih

**Affiliations:** 1 Department of Surgery, Chi Mei Medical Center, Tainan, R.O.C., Taiwan; 2 Department of Pharmacology, National Defense Medical Center, Taipei, R.O.C., Taiwan; 3 Department of Nursing, Kang-Ning Junior College of Medical Care and Management, Taipei, R.O.C., Taiwan; 4 Department of Physiology, National Defense Medical Center, Taipei, R.O.C., Taiwan; 5 Graduate Institute of Medical Sciences, National Defense Medical Center, Taipei, R.O.C., Taiwan; University of Texas Medical Branch, UNITED STATES

## Abstract

Septic shock is a syndrome with severe hypotension and multiple organ dysfunction caused by an imbalance between pro-inflammatory and anti-inflammatory response. The most common risk factor of acute lung injury is severe sepsis. Patients with sepsis-related acute respiratory distress syndrome have higher mortality. Recent studies reveal regulatory roles of Wnt3a and Wnt5a signaling in inflammatory processes. Wnt3a signaling has been implicated in anti-inflammatory effects, whereas Wnt5a signaling has been postulated to have pro-inflammatory properties. However, the balance between Wnt3a and Wnt5a signaling pathway in the lung of rats with endotoxic shock has not been determined. Thus, we investigated the major components of Wnt3a and Wnt5a signaling pathway in the lung of endotoxemic rats. Male Wistar rats were intravenously infused with saline or lipopolysaccharide (LPS, 10 mg/kg). The changes of hemodynamics, biochemical variables, and arterial blood gas were examined during the experimental period. At 6 h after saline or LPS, animals were sacrificed, and lungs were obtained for analyzing superoxide production, water accumulation, histologic assessment, and protein expressions of Wnt3a and Wnt5a signaling pathway. Animals that received LPS showed circulatory failure, multiple organ dysfunction, metabolic acidosis, hyperventilation, lung edema, and high mortality. The lung from rats with endotoxic shock exhibited significant decreases in the levels of Wnt3a, Fzd1, Dsh1, phosphorylated GSK-3β at Ser9, and β-catenin. In contrast, the expressions of Wnt5a, Fzd5, and CaMKII were up-regulated in the lung of endotoxemic rats. These findings indicate the major components of Wnt3a and Wnt5a signaling in the lung are disturbed under endotoxic insult.

## Introduction

Severe sepsis is a syndrome with multiple organ dysfunction caused by systemic inflammation in response to circulating microbes or microbial toxins [1]. Lipopolysaccharide (LPS), a component of Gram-negative bacteria, is responsible for initiating the uncontrolled release of immunoregulatory cytokines from immune cells [2]. Recognition of LPS by Toll-like receptors (TLRs) drives various cellular and subcellular pathways that result in organ damage in sepsis [3]. Despite considerable knowledge and improved intensive care techniques, mortality of septic patients still remains high. Thus, researches into governing effectively these cascades and improving the outcomes of sepsis are urgently needed.

The Wnt pathway is an evolutionarily essential signaling system that controls organ development and homeostasis in the adult [4–6]. Wnt proteins can be exclusively divided into two groups, “canonical” and “non-canonical” Wnts, of which Wnt3a and Wnt5a are the most obvious members [7]. Canonical Wnt3a signaling increases the levels of β-catenin that can induce transcriptional activation of various target genes. Interaction of Wnt3a with Frizzled (Fzd) receptors leads to recruitment and activation of Dishevelled (Dsh) protein. Active Dsh inhibits the activity of glycogen synthase kinase-3β (GSK-3β), and then decreases the proteasomal degradation of β-catenin [7, 8]. In contrast to Wnt3a, non-canonical Wnt5a and Fzd receptors interact to stimulate phospholipase C-mediated release of intracellular Ca^2+^. Increased Ca^2+^ levels enhance calcium/calmodulin-dependent protein kinase II (CaMKII) activity, and then affect different downstream molecules as well as cellular processes [7, 9, 10].

Recent studies reveal regulatory roles of Wnt3a and Wnt5a signaling in infectious and inflammatory processes. Exogenously added Wnt3a can increase β-catenin levels to inhibit pro-inflammatory cytokine production in mycobacteria-infected macrophages [11], suppress vascular cell adhesion molecule-1 expression in hematopoietic cells [12], and block transendothelial migration of human monocytes [13]. Activation of Wnt3a/β-catenin signaling during lung injury also prevents alveolar epithelial cells (AECs) death [14], whereas deletion of β-catenin in AECs reduces cell survival in bleomycin-induced lung injury experiment [15]. Moreover, β-catenin in intestinal dendritic cells is required for the expression of anti-inflammatory mediators, and ablation of β-catenin expression in dendritic cells enhances inflammatory responses in the model of inflammatory bowel disease [16]. These data suggest that Wnt3a/β-catenin signaling possesses anti-inflammatory functions.

Wnt5a has been postulated to be a novel marker of inflammatory diseases with intrinsic pro-inflammatory properties [17]. Elevated Wnt5a levels are observed in different inflammatory conditions such as rheumatoid arthritis, psoriasis, and atherosclerosis [18–20]. In addition, Wnt5a and its receptor Fzd5 are upregulated in human macrophages following stimulation with different mycobacterial species, and are expressed in the lungs of Mycobacterium tuberculosis-infected patients [21]. Pereira et al. has reported that Wnt5a is involved in inflammatory macrophage signaling in sepsis, and knockdown of Wnt5a significantly decreases transcription and secretion of the pro-inflammatory cytokines [22]. Thus, Wnt5a plays an important role in initiating inflammatory responses via signaling through macrophages.

Although previous studies show different roles of Wnt3a and Wnt5a in inflammatory diseases, the changes of canonical Wnt3a and non-canonical Wnt5a signal transduction cascade in the lung of rats with endotoxic shock remain unknown. To characterize the implication of Wnt3a and Wnt5a signal transduction cascade in the systemic response to endotoxin, we compared several aspects of septic pathophysiology and the major components of Wnt3a and Wnt5a signaling pathway in the lung between healthy and endotoxic shock rats. This enabled us to clarify the role of Wnt3a and Wnt5a signaling pathway in endotoxic lung.

## Materials and Methods

### Ethics statement

All animal experiments were approved by the Institutional Animal Care and Use Committee of National Defense Medical Center (Taipei, Taiwan) (Permit Number: IACUC-11-132) and performed in adherence to the National Institutes of Health guidelines for the treatment of animals and ethical animal research. We used humane endpoints and euthanized animals prior to the end of the experiments. Weight loss, inappetence, extreme reluctance to stand, depression coupled with low body temperature, dyspnea, cyanosis, severe diarrhea, seizures, paralysis of one or more extremities were the signs we used to determine when the animals should be euthanized. An overdosed anesthetic (sodium pentobarbital) was the method of euthanasia we used in this study. We observed and monitored the health of the animals every 1 h, and there were no unexpected deaths in these cases. Anesthesia (sodium pentobarbital) was used to reduce the suffering and distress of animals before any procedure that is potentially painful or stressful.

### Animals and experimental protocols

Male Wistar rats were purchased from BioLASCO Taiwan Co (Taipei, R.O.C., Taiwan) and were guaranteed free of particular pathogens. All rats were bred and maintained under a 12 h light-dark cycle at a controlled temperature (21°C ± 2°C) with free access to standard rat chow and tap water. Rats were anesthetized by intraperitoneal injection of sodium pentobarbital (50 mg/kg). The left carotid artery was cannulated and connected to a pressure transducer (P23ID, Statham, Oxnard, CA, USA) to measure mean arterial blood pressure (MAP) and heart rate (HR), which were displayed on a MacLab/4e poly-graph recorder (AD Instruments Pty Ltd., Castle Hill, Australia). The right jugular vein was cannulated for the administration of drugs. After recovering to the normal condition overnight, animals were given vehicle (saline) or *E*. *coli* LPS (10 mg/kg, i.v.) and then monitored for 6 h. During the experimental period, we examined changes in hemodynamics (i.e., MAP, HR, and pressor responses to 1 μg/kg norepinephrine (NE)), blood glucose, hepatic function index (i.e., alanine aminotransferase [ALT]), renal function index (i.e., creatinine [CRE] and blood urea nitrogen [BUN]), cell injury index (i.e., lactate dehydrogenase [LDH]), and arterial blood gas (i.e., HCO_3_^-^, base excess (BE), PaCO_2_, and PaO_2_). Blood samples were obtained at baseline (i.e., time 0) and specified times (i.e., at 1, 2, 4, and 6 h) throughout all procedures. Each volume of blood removed was immediately replaced by the injection of an equal volume of sterile saline. At 6 h after saline or LPS, rats were sacrificed by overdosed pentobarbital, and lungs were obtained for examining superoxide production, water accumulation, histologic assessment, and protein expression of Wnt3a and Wnt5a signaling pathway. The survival rate was calculated during the experimental period.

### Measurement of blood glucose and arterial blood gas

Before the blood sample was centrifuged to prepare serum, ten microliters of whole blood was taken to measure the glucose level in blood by a One Touch II blood glucose monitoring system (Lifescan, Milpitas, CA, USA). One hundred and eighty microliters of whole blood was used to examine the levels of HCO_3_^-^, BE, PaCO_2_, and PaO_2_ by an arterial blood gas analyzer (AVL OPTI Critical Care Analyzer, AVL Scientific Corp., Roswell, GA, USA).

### Biochemical detection

Blood samples were collected from a catheter placed in the carotid artery, and then were immediately centrifuged at 16,000 *g* for 2 min to obtain the serum for measuring biochemical variables. Sixty microliters of serum was used to analyze organ functions at baseline (i.e., time 0) and at 1, 2, 4, and 6 h. All of these biochemical variables were analyzed by Fuji DRI-CHEM 3030 (Fuji Photo Film, Tokyo, Japan). These enzymes measured in the serum were regarded as biochemical indicators of organ function. For instance, liver function was assessed by measuring the serum levels of ALT. Renal function was assessed by measuring the serum levels of CRE and BUN. In addition, LDH was measured to evaluate the extent of organ injury.

### Measurement of superoxide production in the lung

After the lung tissue was removed from the animal, it was cut into 5 x 5 mm pieces and incubated with warmed (37°C), oxygenated (95% O_2_/5% CO_2_) Krebs-HEPES buffer for 10 min. They were gently transferred to 96-well microplates containing 100 microliters of Krebs-HEPES buffer with 50 microliters of lucigenin (1.25 mM), and then the microplates were placed into a microplate luminometer (Hidex Microplate Luminometer, Turku, Finland). Counts were obtained in duplicate at 60-sec intervals. The lung tissue was dried in a 95°C oven for 24 h. The result was expressed as count per second per milligram of organ dry weight.

### Determination of lung water accumulation

The lung water accumulation was used as an index of lung edema. Rats were sacrificed by overdosed pentobarbital, and the right lung weight was measured after its excision (wet weight). The right lung tissue was then dried in an oven at 50°C for 2 weeks and reweighed as dry weight. The right lung water accumulation was calculated by subtracting the dry weight from the wet weight, and then normalized by rat body weight.

### Histologic assessment

The lungs were harvested at 6 h and fixed in buffered formaldehyde (10% in phosphate-buffered saline, pH 7.4). The fixed organs were embedded in paraffin and stained with the hematoxylin and eosin reagent for light microscopy. Histologic alternation was quantitatively as an index of the severity of polymorphonuclear neutrophil (PMN) infiltration in the lung. Each tissue section was examined under high-power fields, and a score from 0 to 4 was given for each section determined by a pathologist in a blinded fashion.

### Western blot analysis

At the end of *in vivo* experiment, lungs were obtained from animals. Cytosolic, nuclear, and membrane-containing fractions to determine the expressions of Wnt canonical or noncanonical signaling molecules in the lung were obtained by a CNM compartmental protein extraction kit (BioChain Inc., San Francisco, CA, USA) according to the manufacturer’s instruction. Cytosol-containing fractions were used to determine the expressions of Wnt3a, Wnt5a, GSK-3β, CaMKII, and total β-catenin. Nuclear-containing fractions were used to determine the expressions of nuclear β-catenin. Membrane-containing fractions were used to determine the expressions of Fzd and Dsh proteins. Protein concentration was determined by BCA Protein Assay Kit (Thermo scientific, Rockford, IL, USA). Samples containing 100 μg of protein were processed for analysis. Protein was subjected to 10% sodium dodecyl sulfate-polyacrylamide gel electrophoresis under reducing condition. The protein was transferred onto nitrocellulose membranes. The membranes were blocked with 5% non-fat milk in Tris buffer solution containing 0.1% Tween-20 (TBST) for 1.5 h at room temperature, and then incubated overnight at 4°C, with gentle shaking with primary antibody (Wnt3a, 1:1000; Fzd1, 1:1000; Dsh1, 1:1000; total-GSK-3β, 1:8000; phospho-GSK-3β at Ser9, 1:3000; total β-catenin, 1:8000; nuclear β-catenin, 1:5000; Wnt5a, 1:1000; Fzd5, 1:1000; total CaMKII, 1:500; phospho-CaMKII at Thr286, 1:1000; β-actin, 1:10000; Histone H3, 1:5000; all purchased from Abcam, Cambridge, UK, except Histone H3 from GeneTex, Irvine, California, USA) in TBST buffer with 3% milk. The membranes were washed and then incubated at room temperature with horseradish peroxidase-conjugated goat anti-mouse IgG (BD Transduction Laboratories, Lexington, KY, USA) or horseradish peroxidase-conjugated goat anti-rabbit IgG (Cell signaling Technology Inc, Danvers, MA, USA) in TBST buffer. After washed, the proteins were visualized by using enhanced chemiluminescence Western blotting detection reagent (Thermo scientific, Rockford, IL, USA). Bands were quantified by ImageJ software version 1.46r (National Institutes of Health, Bethesda, MD, USA).

### Statistical analysis

We used Graphpad Prism software version 5.0 (Graphpad Prism software, Inc., La Jolla, CA, USA) to analyze all data. Results are expressed as mean ± standard error of mean of *n* determinations, where *n* represents the number of animals studied. Statistical significance was determined through the unpaired Student’s t-test. In addition, the chi-square test (Fisher’s exact test) was used to determine significant differences in survival rates of Control and LPS groups. A *p* value <0.05 was considered statistically significant.

## Results

### Changes of hemodynamic parameters, organ functions, and survival rate in endotoxemic rats

**Fig 1:**
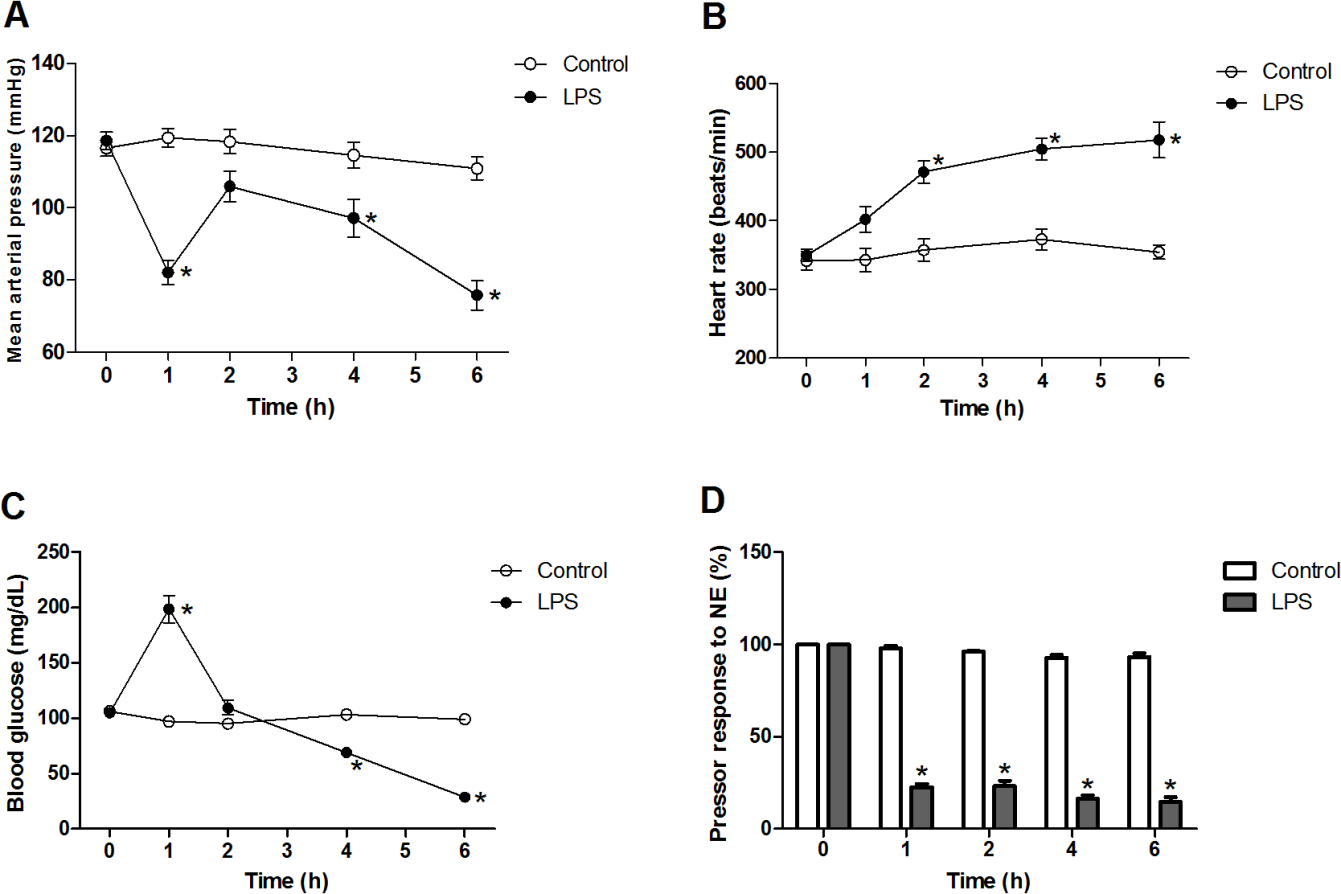
Changes of (A) mean arterial pressure (MAP), (B) heart rate (HR), (C) blood glucose, and (D) pressor response to norepinephrine (NE) in rats treated with lipopolysaccharide (LPS). Depicted are changes in MAP, HR, blood glucose, and pressor response to NE (1 μg/kg, i.v.) during the experimental period in rats that received saline (Control; 0.9% NaCl, i.v., *n* = 10) or LPS (10 mg/kg, i.v., *n* = 10). Data expressed as mean ± SEM. *P < 0.05, LPS vs. Control.

The baseline values for MAP, HR, blood glucose, pressor responses to NE, and biochemical variables were not different between LPS and Control group (Fig 1 and Table 1). The animals that received LPS showed significant decreases in MAP, blood glucose, and pressor responses to NE at the end of the experiment (Fig 1A, 1C and 1D). By contrast, a significant increase in HR was observed at 6 h after LPS (Fig 1B). In addition, LPS elicited significant increases in ALT, BUN, CRE, and LDH levels during the experimental period (Table 1). The survival rate of animals was 83% at 2h, 80% at 4h, and 33% at 6h after administration of LPS (Table 2). However, in the Control group, all hemodynamics and organ function indexes were not significantly changed and no mortality was observed during the experimental period (Fig 1 and Table 1). Thus, the injection of LPS caused severe hypotension, tachycardia, hypoglycemia, vascular hyporeactivity, multiple organ dysfunction syndrome, and high mortality at the end of the experiment.

**Table 1:** Changes of biochemical parameters during the experimental period.

	0 h	1 h	2 h	4 h	6 h
**ALT (U/L)**					
Control	24.20 ± 2.05	21.90 ± 2.27	19.60 ± 2.12	18.60 ± 1.73	18.50 ± 2.07
LPS	19.30 ± 1.84	29.70 ± 3.53	35.50 ± 5.07	78.10 ± 12.53*	209.20 ± 38.77*
**BUN (mg/dL)**					
Control	18.45 ± 1.51	17.39 ± 1.43	16.40 ± 0.96	16.60 ± 1.04	17.47 ± 1.50
LPS	20.48 ± 0.97	30.27 ± 2.13*	37.89 ± 2.47*	57.44 ± 3.14*	75.53 ± 4.81*
**CRE (mg/dL)**					
Control	0.20 ± 0	0.20 ± 0	0.20 ± 0	0.20 ± 0	0.20 ± 0
LPS	0.20 ± 0	0.29 ± 0.04	0.31 ± 0.04	0.49 ± 0.06*	0.78 ± 0.11*
**LDH (U/L)**					
Control	102.90 ± 9.41	85.10 ± 5.33	73.00 ± 4.24	69.20 ± 4.32	85.80 ± 8.48
LPS	96.80 ± 7.65	277.00 ± 39.23	348.90 ± 31.86	1467.20 ± 318.38*	3649.00 ± 543.12*

Depicted are changes in alanine aminotransferase (ALT), blood urea nitrogen (BUN), creatinine (CRE), and lactate dehydrogenase (LDH) during the experimental period in rats that received saline (Control; 0.9% NaCl, i.v., n = 10) or LPS (10 mg/kg, i.v., n = 10). Data expressed as mean ± SEM.

*P < 0.05, LPS vs. Control.

**Table 2:** Changes of survival rate during the experimental period.

Groups	1-h Survival Rate (%)	2-h Survival Rate (%)	4-h Survival Rate (%)	6-h Survival Rate (%)
Control	100 (10/10)	100 (10/10)	100 (10/10)	100 (10/10)
LPS	100 (30/30)	83 (25/30)	80 (24/30)	33 (10/30)*

Depiction of the changes in survival rate during the experimental period in different groups of animals which received saline (Control, 0.9% NaCl, i.v., n = 10) or LPS (10 mg/kg, i.v., n = 30). Data expressed as percentage of rats survived at the observed time point.

*P < 0.05, LPS vs. Control.

### Changes of arterial blood gas in endotoxemic rats

**Fig 2:**
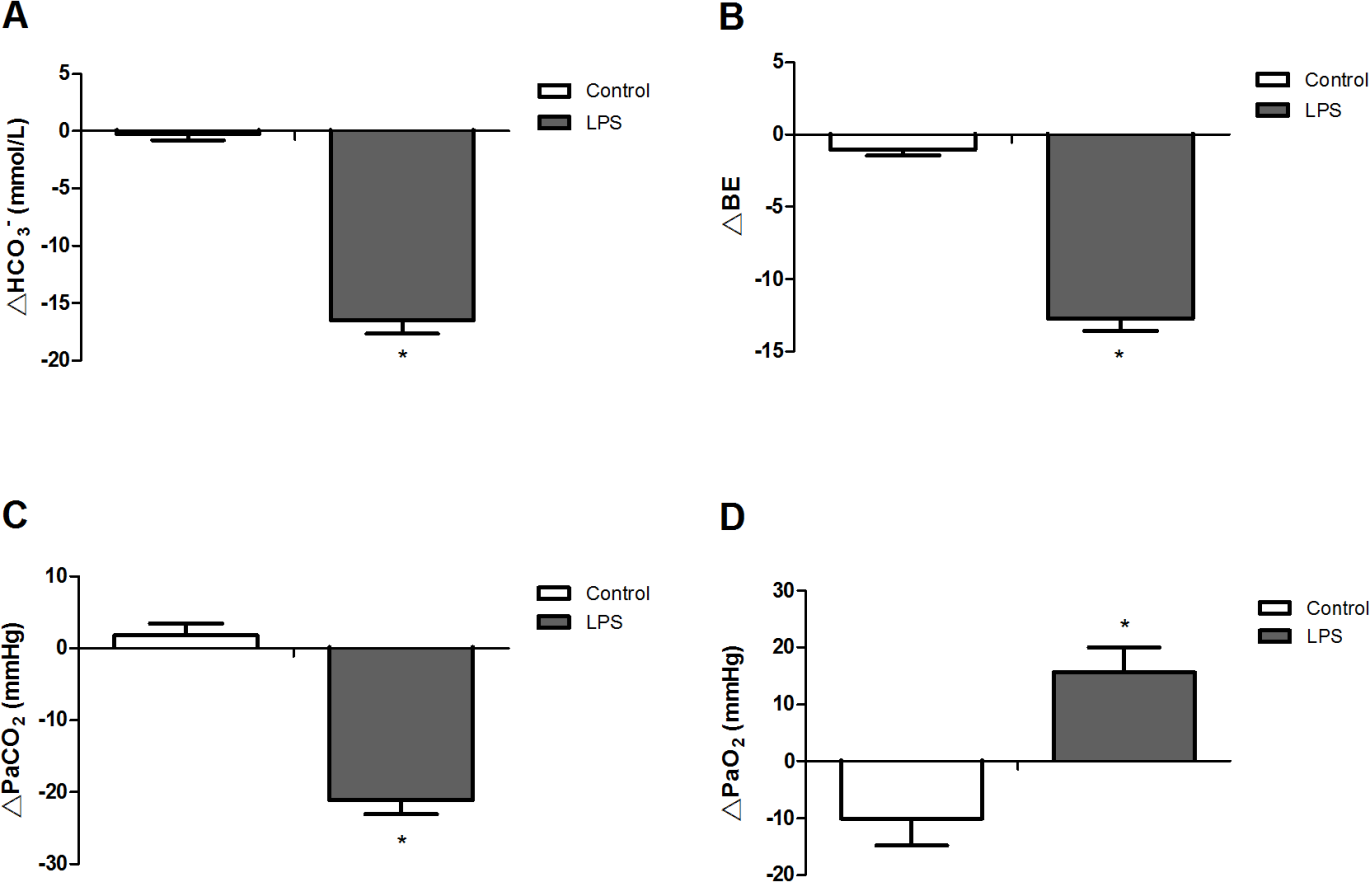
Changes of blood (A) HCO3-, (B) base excess (BE), (C) PaCO_2_, and (D) PaO_2_ in rats treated with lipopolysaccharide (LPS). Depicted are changes in HCO_3_^-^, BE, PaCO_2_, and PaO_2_ at the end of experiment (at 6 h) in rats that received saline (Control; 0.9% NaCl, i.v., n = 8) or LPS (10 mg/kg, i.v., n = 8). The values of all groups are normalized at 0 h as zero. Data expressed as mean ± SEM. *P < 0.05, LPS vs. Control.

To examine the changes of acid-base balance and lung function in LPS-induced endotoxemia animals, we measured the levels of HCO_3_^-^, BE, PaCO_2_, and PaO_2_. LPS led to significant reductions in HCO_3_^-^ and BE levels at the end of the experiment (Fig 2A and 2B). In addition, rats with endotoxic shock exhibited a decrease in PaCO_2_ level, and an increase in PaO_2_ level at 6h after LPS treatment (Fig 2C and 2D). However, there were no significant changes in HCO_3_^-^, BE, PaCO_2_, and PaO_2_ levels in the arterial blood at 6h after LPS treatment in the Control group. These results indicate that administration of LPS elicits metabolic acidosis and hyperventilation at the end of the experiment.

### Changes of superoxide production, water accumulation and neutrophil infiltration in the lung from rats with endotoxic shock

**Fig 3:**
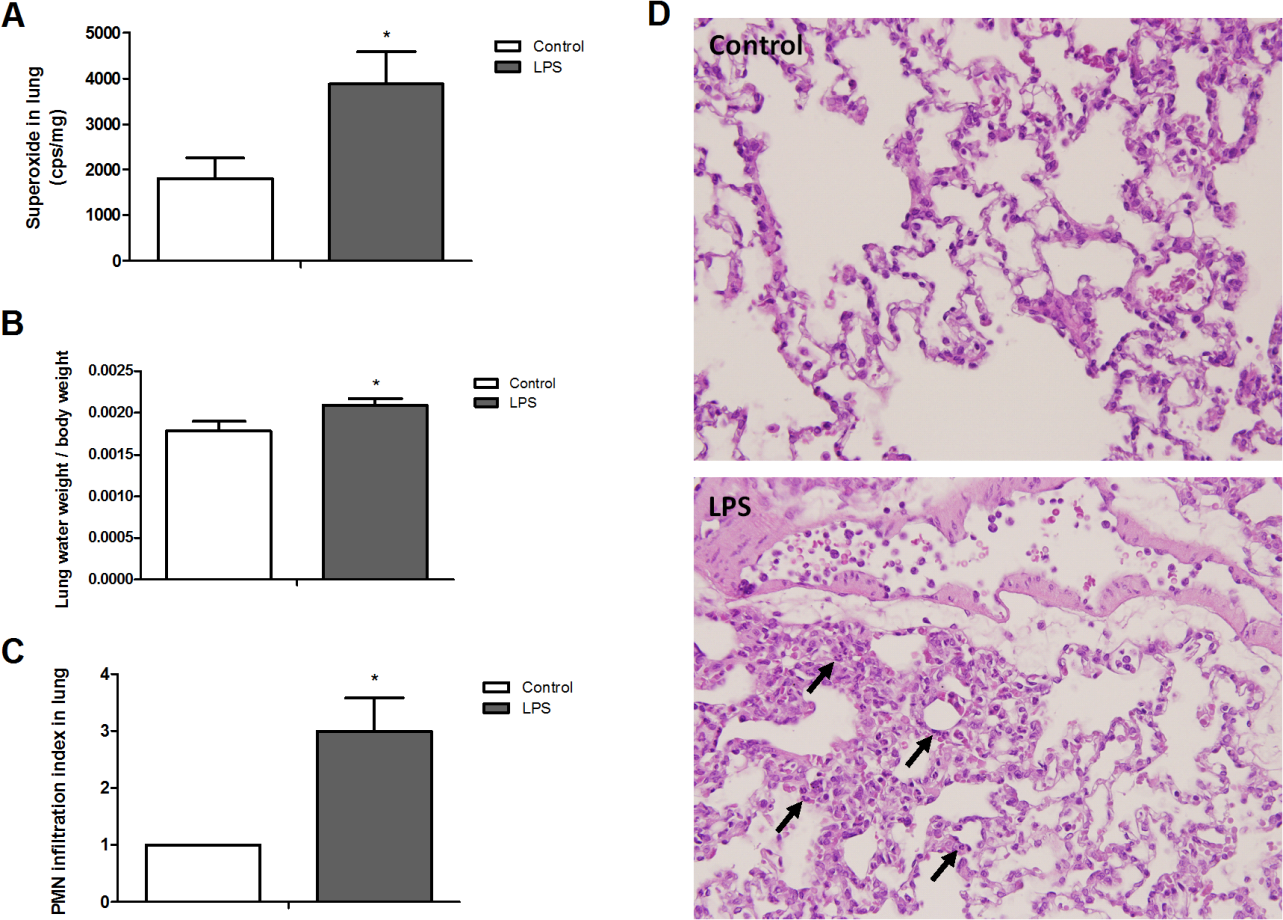
Changes of lung (A) superoxide production and (B) water accumulation in rats treated with lipopolysaccharide (LPS). Depicted are changes in superoxide production and water accumulation at the end of experiment (at 6 h) in rats that received saline (Control; 0.9% NaCl, i.v., n = 7) or LPS (10 mg/kg, i.v., n = 7). Data expressed as mean ± SEM. *P < 0.05, LPS vs. Control. **(C) Polymorphonuclear neutrophil (PMN) infiltration index and (D) representative histopathologic features of lung tissue sections obtained from rats treated with LPS.** Depiction of the changes in PMN infiltration index at the end of experiment (at 6 h) in different groups of animals which received saline (Control, 0.9% NaCl, i.v., n = 3) or LPS (10 mg/kg, i.v., n = 3). Data expressed as mean ± SEM. *P < 0.05, LPS vs. Control. Light microscopy showed lung sections of rats in Control and LPS group. Arrows represent neutrophil infiltration and interstitial edema. Original magnification x 400.

To investigate the degrees of lung inflammation and edema in LPS-induced endotoxemia animals, we measured the levels of superoxide production, water accumulation and neutrophil infiltration in the lung. The lung from rats with endotoxic shock revealed significant increases in superoxide level and water accumulation when compared with the lung from Control group at 6h after LPS treatment (Fig 3A and 3B). In addition, LPS led to marked interstitial edema and overt neutrophil infiltration in the lung at the end of the experiment (Fig 3C and 3D). Thus, the injection of LPS caused significant inflammation and edema in the lung of rats with endotoxic shock.

### Changes of Wnt3a signaling pathway in the lung from rats with endotoxic shock

**Fig 4:**
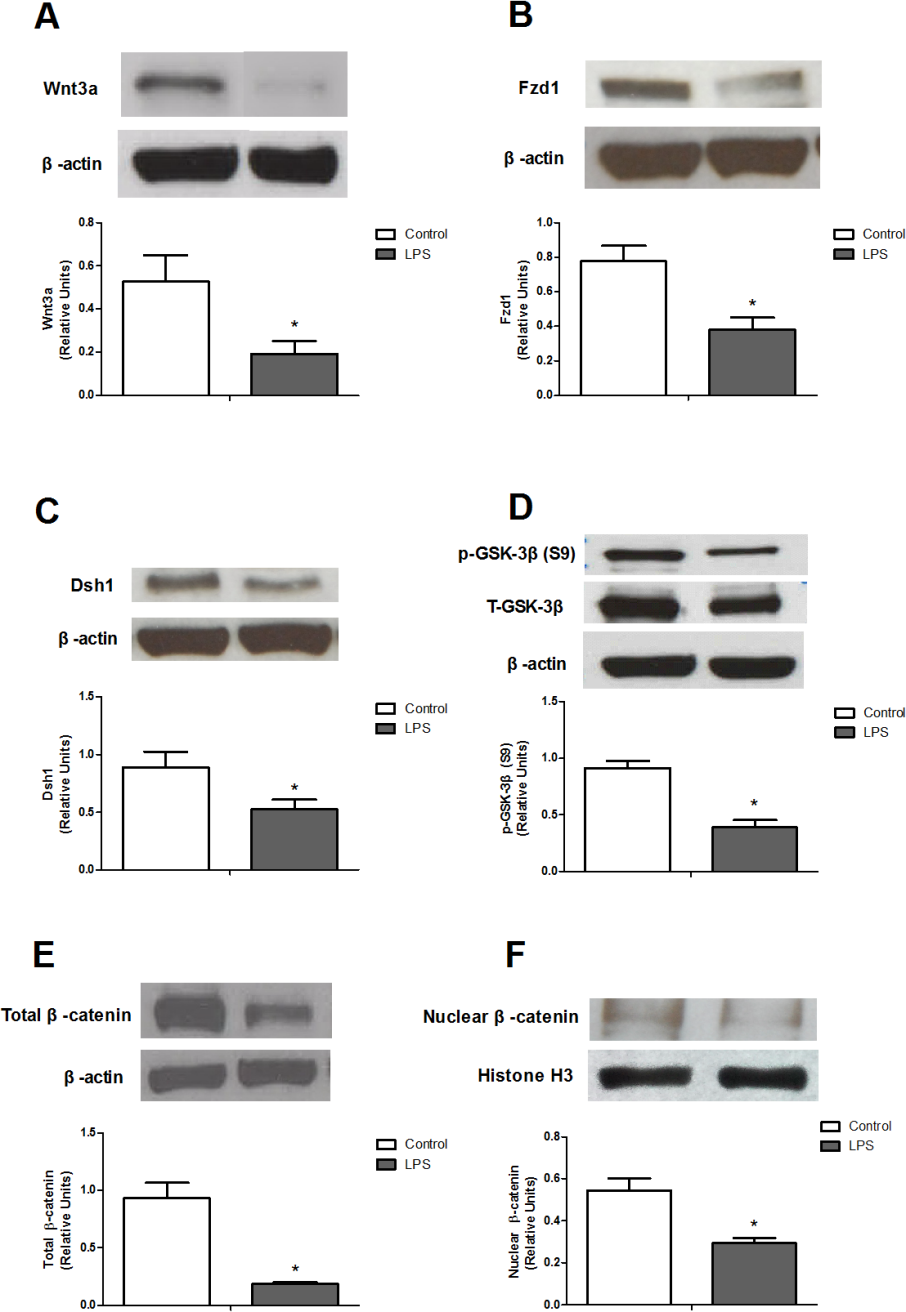
Protein expressions of lung (A) Wnt3a, (B) Frizzled1 (Fzd1), (C) Dishevelled1 (Dsh1), (D) phosphorylated glycogen synthase kinase-3β (GSK-3β), (E) total β-catenin, and (F) nuclear β-catenin in rats treated with lipopolysaccharide (LPS). The lung homogenates from rats in the presence or absence of LPS (10 mg/kg, i.v.) were used to measure the levels of Wnt3a (n = 6), Fzd1 (n = 6), Dishevelled-1 (n = 5), phosphorylated GSK-3β at Ser9 (n = 4), total β-catenin (n = 5), and nuclear β-catenin (n = 4). Data expressed as mean ± SEM. *P < 0.05, LPS vs. Control.

To study whether the major components of Wnt3a signaling pathway were affected in the lung of endotoxemic rats, we performed Western blotting to measure the levels of Wnt3a, Fzd1, Dsh1, phosphorylated GSK-3β at Ser9, total β-catenin, and nuclear β-catenin. Our results showed that the protein expression of Wnt3a, Fzd1, Dsh1, phosphorylated GSK-3β, total β-catenin, and nuclear β-catenin were detectable in lung homogenates obtained from the Control group (Fig 4A–4F). However, lung homogenates from rats that received LPS exhibited significant decreases in the levels of Wnt3a, Fzd1, Dsh1, phosphorylated GSK-3βat Ser9, total β-catenin, and nuclear β-catenin (Fig 4A–4F). Thus, the major components of Wnt3a signaling pathway in the lung were downregulated in endotoxemic rats.

### Changes of Wnt5a signaling pathway in the lung from rats with endotoxic shock

**Fig 5:**
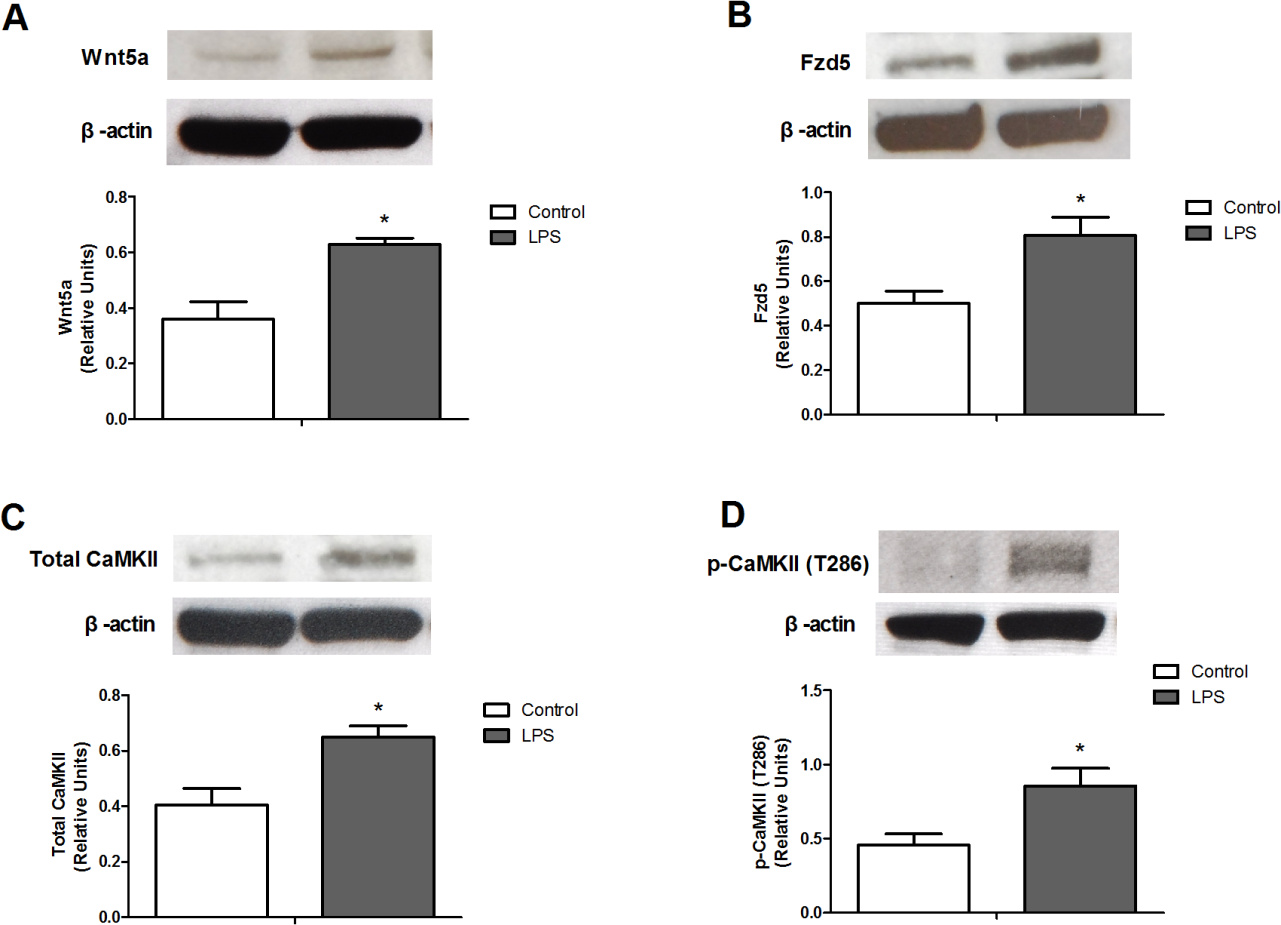
Protein expressions of lung (A) Wnt5a, (B) Frizzled5 (Fzd5), (C) total calcium/calmodulin-dependent protein kinase II (CaMKII), and (D) phosphorylated CaMKII in rats treated with lipopolysaccharide (LPS). The lung homogenates from rats in the presence or absence of LPS (10 mg/kg, i.v.) were used to measure the levels of Wnt5a (n = 4), Fzd5 (n = 4), total CaMKII (n = 4), and phosphorylated CaMKII (n = 4). Data expressed as mean ± SEM. *P < 0.05, LPS vs. Control.

To investigate whether the major components of Wnt5a signaling pathway were affected in the lung of endotoxemic rats, we measured the protein expression of Wnt5a, Fzd5, total CaMKII, and phosphorylated CaMKII at Thr286. The levels of Wnt5a, Fzd5, total CaMKII, and phosphorylated CaMKII in the lung from rats with endotoxic shock were significantly increased when compared with the Control group (Fig 5A–5D). These data indicate the major components of Wnt5a signaling pathway in the lung are up-regulated in endotoxemic rats.

## Discussion

In this study, we investigated the signal transduction cascade of canonical Wnt3a and non-canonical Wnt5a signaling in the lung from rats with endotoxic shock. Animals that received LPS exhibited circulatory failure, multiple organ dysfunction syndrome, metabolic acidosis, hyperventilation, and high mortality. LPS also caused lung inflammation and edema at the end of the experiment. We found the lung from rats with endotoxic shock exhibited significant decreases in the levels of Wnt3a, Fzd1, Dsh1, phosphorylated GSK-3β at Ser9, total β-catenin, and nuclear β-catenin when compared with the Control group. In contrast, the expression of Wnt5a, Fzd5, total CaMKII, and phosphorylated CaMKII at Thr286 were up-regulated in the lung of endotoxemic rats. These findings suggest that imbalance between Wnt3a and Wnt5a signaling pathway occurs in the lung of rats with endotoxic shock.

Acute lung injury occurs with a high incidence and has a substantial impact on public health in the world. Severe sepsis is the most common risk factor for acute lung injury in a large prospective cohort study [23]. Sepsis-related acute respiratory distress syndrome (ARDS) has poorer recovery from lung injury and higher mortality than non-sepsis-related ARDS [24]. Therefore, it is important to understand more about the detailed mechanisms of sepsis-related acute lung injury. In order to induce sepsis-related acute lung injury in this study, we selected *E*. *coli* LPS to be a key pathogen recognition molecule. Indeed, our results showed rats that received LPS manifested clinical septic signs and lung dysfunction, as seen in patients with severe sepsis. In addition, increased superoxide production, neutrophil infiltration, and water accumulation were observed in the lung from rats with endotoxic shock. These data indicate this model can help us understand complex mechanisms that involved in eondotoxemia-related lung injury.

Wnt3a/β-catenin signaling has recently been implicated in the modulation of inflammatory processes. Neumann et al. demonstrates that Mycobacterium tuberculosis infection lowers β-catenin levels and increases TNF release in murine macrophages. Addition of Wnt3a increases β-catenin stabilization and reduces TNF release in macrophages infected with Mycobacterium tuberculosis [11]. Thus, it is likely that Wnt3a/β-catenin signaling contributes to the beneficial effects on cell inflammation and death during stress conditions. Indeed, our study showed that the expression of Wnt3a and β-catenin were significantly down-regulated in the lung of LPS-induced sepsis animals, indicating that the reduction of Wnt3a and β-catenin in the lung could be associated with lung inflammation and injury in endotoxemia.

The canonical Wnt3a/β-catenin pathway is activated by Wnt3a binding to a member of the Fzd receptor family [25]. Dsh is a key component of several signaling pathways that are initiated by Wnt and Fzd receptors [26]. The recruitment of Dsh to Fzd receptor triggers the dissociation of a β-catenin degradation complex, resulting in β-catenin stabilization. In order to examine whether the downstream components of Wnt3a signaling were affected during endotoxemia, the expression of Fzd1 and Dsh1 in the lung were analyzed. Our results revealed that LPS treatment reduced the levels of Fzd1 and Dsh1 in the lung, similar to the changes of Wnt3a and β-catenin. This is the first study demonstrating that the expression of Fzd1 and Dsh1 were down-regulated in the lung of endotoxic animals. Decreased Fzd1 and Dsh1 may hinder the sensitivity of Wnt3a/β-catenin signaling on regulating inflammatory responses. Therefore, future studies would determine the outcomes after the intervention for maintaining the homeostasis of Fzd1 and Dsh1 in sepsis.

Several transcription factors such as nuclear factor-kappa B and β-catenin can be regulated by GSK-3β [27, 28]. Active Dsh inhibits the activity of GSK-3β, and then results in the accumulation of β-catenin. We found that administration of LPS suppressed the phosphorylation levels of GSK-3β at Ser9 residue as well as the levels of β-catenin. Phosphorylation of GSK-3β at Ser9 is known to inactivate its activity [29]. This fits well with some studies in endotoxemia model where the protective effects of GSK-3β inhibitors on cytokine production and organ dysfunction were identified [30, 31]. Despite GSK-3β inhibitor is recognized as a potential treatment of sepsis, our data demonstrated that many components of canonical Wnt3a signaling were also affected in the lung of rats with endotoxic shock. Thus, further studies to clarify the therapeutic effects of different Wnt3a signal enhancers on septic shock are needed.

In contrast to Wnt3a signaling, Wnt5a signaling serves as a novel pro-inflammatory marker in infectious and inflammatory processes [32]. TLR agonists can enhance the expressions of Wnt5a and its receptor Fzd5 in human macrophage, which is the key factor in systemic inflammatory response [21, 22]. However, it is unclear which tissue is the major source of Wnt5a. In the present study, we measured the key components of Wnt5a signaling pathway in the lung of endotoxemia rats to gain further insights into Wnt5a/Fzd5/CaMKII biology. Our results exhibited that Wnt5a expressed more in the lung of rats with endotoxic shock, which is consistent with a recent study, showing that lungs from septic animals and patients have strong immunohistochemistry intensity of Wnt5a [33]. In addition, the activation of CaMKII is dependent on phosphorylation at Thr286 [34]. We provided further evidence that the expression of Fzd5, total CaMKII, and phosphorylated CaMKII at Thr286 were enhanced in endotoxic lungs. These data indicate that the major components of Wnt5a signaling pathway in the lung are disturbed under endotoxic conditions.

In conclusion, our findings demonstrate complementary patterns of canonical Wnt3a and non-canonical Wnt5a signal transduction cascade in the lung of rats with endotoxic shock. The deteriorations in Wnt3a/Fzd1/β-catenin and Wnt5a/Fzd5/CaMKII signaling pathways could be biomarkers of lung injury in this endotoxemic model. Understanding these detailed regulation pathways of the inflammatory responses in the lung would be helpful in the design of novel therapies to prevent lung injury in endotoxemia.
